# Sample Preparation Approaches for Determination of Quinolones in Aqueous Matrixes: Systematic Review

**DOI:** 10.1021/acsmeasuresciau.4c00056

**Published:** 2024-11-14

**Authors:** Tainara Aparecida Nunes Ribeiro, Daiane Dulcileia Moraes de Paula, Marcella Matos Cordeiro Borges, Leandro Augusto Calixto, Keyller Bastos Borges

**Affiliations:** † Instituto de Física e Química, Universidade Federal de Itajubá (UNIFEI), Av. BPS, 1303, Pinheirinho, 37500-903 Itajubá, Minas Gerais, Brazil; ‡ Departamento de Ciências Naturais, Universidade Federal de São João del-Rei (UFSJ), Campus Dom Bosco, Praça Dom Helvécio 74, Fábricas, 36301-160 São João del-Rei, Minas Gerais, Brazil; § Departamento de Ciências Farmacêuticas, Instituto de Ciências Ambientais, Químicas e Farmacêuticas, Universidade Federal de São Paulo (UNIFESP), Campus Diadema, Prof. Artur Riedel, 275, Eldorado, 09972-270 Diadema, São Paulo, Brazil

**Keywords:** systematic review, quinolones, fluoroquinolones, sample preparation, liquid−liquid extraction, solid-phase extraction, instrumental analysis, aqueous matrixes

## Abstract

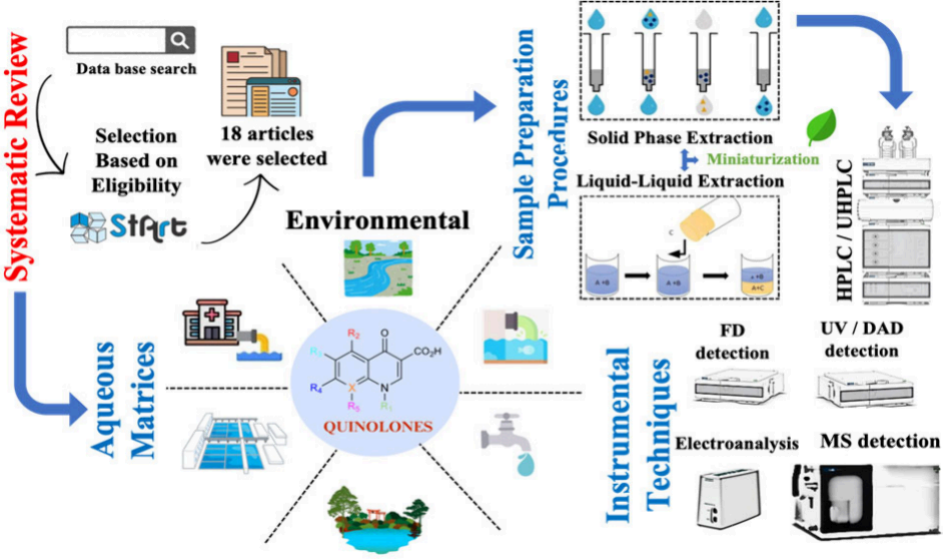

Quinolones and fluoroquinolones are among the most used antibiotics worldwide. Antibiotic resistance genes can be acquired by human pathogens from ambient microorganisms, which can lead to a treatment failure for bacterial infections. Furthermore, determining the levels of quinolones and fluoroquinolones in aqueous matrixes is crucial for safeguarding both human health and the environment. Using sample preparation techniques is essential since these compounds are commonly present in aqueous matrixes at trace levels. Therefore, we aimed to investigate the main analytical methods for the determination of quinolones in aqueous samples based on a systematic literature review. We only considered studies that presented more robust analytical techniques that allowed for more accurate and precise measurement of quinolones/fluoroquinolones in aqueous matrixes. In total, 18 articles met the inclusion criteria and were used in our analysis. A total of 21 quinolone antibacterial agents were investigated in water samples from 13 countries, showing a potential risk around the world. Ciprofloxacin (72.2%), enrofloxacin (61.1%), norfloxacin (50%), marbofloxacin, and levofloxacin (33.3%) were the most frequently evaluated compounds. High-Performance Liquid Chromatography stands out as the predominant instrumental technique for the separation and identification of these compounds. Additionally, among the selected studies in this review, 44% employed liquid–liquid extraction techniques and its miniaturized versions, while 56% opted for solid-phase extraction and its miniaturized variations. Finally, it is important to note that the final method efficiency relies on the entire process, from selecting the instrumental technique with appropriate detection limits, going through the entire sample preparation, to achieving a good recovery through adjustments in the extraction parameters to archive the determination of trace levels.

## 1 Introduction

Antibacterial agents constitute a class of pharmaceuticals that act by inhibiting bacterial growth and causing bacterial destruction. They can be categorized as natural, semisynthetic, and synthetic, each differing in their pharmacological, physical, and chemical properties, as well as in their mechanism of action.^1^ Quinolones or 4-quinolones, a diverse group of compounds, exert their action by inhibiting bacterial enzymes such as DNA gyrase and topoisomerase IV. These enzymes play a pivotal role in bacterial replication, leading to the blockage of nucleic acid synthesis.^2^

**Figure 1:**
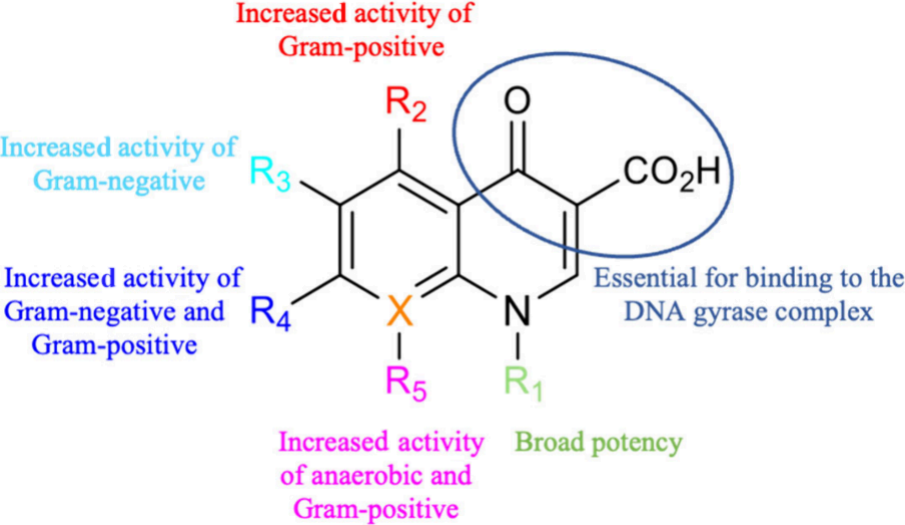
General molecular structure and properties of quinolones.

Quinolones originated from nalidixic acid, considered the pioneering antibiotic in this group.^1^ Numerous structural and substituent modifications have been introduced at positions N-1, C-5, C-6, C-7, and C-8, aiming to broaden the spectrum of action and enhance the chemical, pharmacokinetic, and pharmacological properties (Figure 1). Notably, fluoroquinolones, developed through fluorine substitution at position 6, exhibit activity against Gram-negative and Gram-positive microorganisms, as well as chlamydia and mycoplasmas.^3,4^

Quinolones and fluoroquinolones are crucial antimicrobials in medicine, utilized in clinical or veterinary treatments, and available as racemates or in enantiomerically pure forms. However, according to the World Health Organization (WHO), their administration should be optimized to maximize therapeutic efficacy and minimize the risk of resistance, a pervasive global issue.^5^ Bacterial resistance is a natural phenomenon stemming from selective pressure associated with bacterial mechanisms and genetic evolution. The WHO has classified it as one of the top 10 public health problems due to the indiscriminate use of antibacterial, exacerbating this challenge.^6−9^

In this context, it is noteworthy that quinolone antibiotics are frequently found in the environment, with total fluoroquinolone loads ranging from 0.3 to 8.1 g day^–1^ for small wastewater treatment plants, to 190 to 326 g day^–1^ in cities such as Zagreb and Zurich, and even up to 216 to 1228 g day^–1^ in urban Chinese wastewater.^10^ A study conducted across European cities revealed that normalized ciprofloxacin loads in water ranged from a minimum average of 37.6 to 409.9 mg per day^–1,000^ people^–1^ and 4.3 to 727.4 mg per day^–1,000^ people^–1^ for ofloxacin.^11^

Treatments for bacterial infections can fail, because human pathogens can acquire antibiotic resistance genes (ARGs) from ambient microorganisms. This situation raises concerns about resistance, as significant correlations have been identified between fluoroquinolones and plasmid-mediated quinolone resistance genes (qnrD, oqxB, qepA, qnrS, and oqxA) in wastewater and soil samples near pig farms.^12^ Additionally, resistance to arsenic and ciprofloxacin has been observed in hospital effluents.^13^ Studies have also indicated concentrations of ciprofloxacin/ofloxacin and copy numbers of antibiotic resistance genes with reduced susceptibility to fluoroquinolones (qnrS) in hospital wastewater and wastewater treatment plants in Spain and Milan^11,14^ as well as ciprofloxacin and qnrB in hospital wastewater in Singapore.^15^ In addition, one well-known source of ARGs is the aquaculture industry, which is contaminated by the overuse of antibiotics. In addition to quinolone-resistance genes, enrofloxacin administration (only veterinary use) raised the relative abundance of several ARGs and caused ARG dispersion in the sediment bacteria and crayfish stomach.^16^

Therefore, quinolone and fluoroquinolone determination in aqueous matrixes is of paramount importance for environmental protection and human health.^17^ Given the presence of quinolones/fluoroquinolones in water typically at trace concentrations, the implementation of sample preparation procedures is crucial. This process aims to isolate and concentrate these analytes, removing interferents that could compromise the integrity of the analytical results. Thus, sample preparation plays a pivotal role in enhancing the method sensitivity and the reliability of obtained data. Moreover, it enables the attainment of detection limits that comply with the standards required for precise and accuracy analysis.^17,18^

The sample preparation step is often the most resource-intensive due to its time-consuming nature, and it stands as one of the primary sources of error in chemical analysis. Therefore, selecting the most appropriate method involves a consideration of the physicochemical properties of the sample, analysis objectives, and analytical instrument. The literature encompasses various techniques and sample preparation methods, with classical approaches including liquid–liquid extraction (LLE), looking for greener solvents,^19^ and solid-phase extraction (SPE), searching for more advanced nanomaterials,^20^ but recent environmental appeals have demanded its replacement by miniaturization of sample preparation techniques, which are less aggressive (greener sample preparation) to the environment and the analyst.^21^ Among several miniaturized techniques, the following stand out: solid-phase microextraction (SPME), microextraction by packed sorbent (MEPS), stir bar sorptive extraction (SBSE), magnetic solid-phase extraction (MSPE), liquid-phase microextraction (LPME), hollow fiber liquid-phase microextraction (HF-LPME), direct immersion single drop microextraction (DI-SDME), headspace single drop microextraction (HS-SDME, and dispersive liquid–liquid microextraction, etc.^22−24^

Sample preparation optimization is crucial for obtaining precise and accurate results, emphasizing the need for speed, cost-effectiveness, and compatibility with the analytical system.^22^ Thus, this review aimed to provide readers with a systematic overview of methods for the analysis of quinolone antibacterials in aqueous samples. Articles were selected based on inclusion and exclusion criteria to identify studies that conducted the sample preparation procedure and method validation. The data were organized into three tables: (i) summary of sample preparation and analytical methods, (ii) optimization of parameters involved in sample preparation across various studied techniques, and (iii) main information on the validation of methods. Following the compilation of results, data were analyzed, and comparisons were made to assess which sample preparation techniques and analytical methods offered greater robustness and enabled the quantification of quinolones in aqueous matrixes with higher precision and accuracy.

## 2 Methods

A systematic review has been carried out following the guidelines of the Transparent Reporting of Systematic Reviews and Meta-Analyses (PRISMA statement).^25^ The following question of this review is, “What methods of sample preparation of quinolones in water matrixes are described in the literature?”. Three databases included were PubMed/Medline, Scopus, and Web of Science. The timeline was restricted from January 1, 2011, to September 14, 2023. The search strategy was reported at Table 1 and a computational tool (StArt) was used to support trial data.^26^ It is important to mention that some studies may not be found with the software.

**Table 1:** Search Strategy of Databases of Present Systematic Review

Databases	Search strategy (Boolean Descriptors and Operators)
Medline/PubMed	(Sample preparation AND fluoroquinolones OR quinolones OR ciprofloxacin OR enoxacin OR norfloxacin OR lomefloxacin OR clinafloxacin OR gatifloxacin OR moxifloxacin OR levofloxacin OR ofloxacin (title)) AND (determination OR analysis AND water) (title/abstract)
Web of Science	(Sample preparation (abstract)) AND (fluoroquinolones OR quinolones OR ciprofloxacin OR enoxacin OR norfloxacin OR lomefloxacin OR clinafloxacin OR gatifloxacin OR moxifloxacin OR levofloxacin OR ofloxacin (abstract)) AND (determination OR analysis AND water (abstract))
SCOPUS	(Sample preparation AND fluoroquinolones OR quinolones OR ciprofloxacin OR enoxacin OR norfloxacin OR lomefloxacin OR clinafloxacin OR gatifloxacin OR moxifloxacin OR levofloxacin OR ofloxacin (title, abstract, keywords)) AND ((determination OR analysis AND water (title, abstract, keywords))

### 2.1 Eligibility Criteria

#### 2.1.1 Inclusion Criteria

(1) Studies that evaluated specifically water samples; (2) Techniques of liquid–liquid extraction; (3) Techniques of solid-phase extraction; (4) Studies that involved sample preparation; (5) Articles that analyze only fluoroquinolones or quinolones antibiotics; (6) Protocols based on validated analytical methods; and (7) Articles written in English.

#### 2.1.2 Exclusion Criteria

(1) Reviews/dissertation/thesis/book chapter; (2) Studies that do not focus on sample preparation; (3) Studies that analyze food samples and biological fluids; (4) Studies that analyze a mixture of antibiotics different than quinolones or fluoroquinolones; (5) Studies that have not validated analytical methods; (6) Not full text available.

### 2.2 Study Selection

The initial step involved importing articles into the StArt tool from selected databases. Duplicate articles were systematically removed. The subsequent selection process adhered to predefined inclusion and exclusion criteria, which encompassed a thorough examination of the article’s title, keywords, and abstract by two independent authors (D.D.M.P. and T.A.N.R.). Any disparities in their assessments were resolved through discussion. Afterward, the full texts of the selected articles underwent a more detailed assessment, and any articles that did not align with the predefined criteria were excluded from the study.

### 2.3 Data Extraction

The data extracted from the included articles were systematically collected and organized into tables, containing information regarding the analytical state (liquid, aqueous, solid), the types of environmental matrixes studied, and the employed methods and parameters associated with the analytical techniques used. The quality of the studies was assessed by selecting articles that employed validated analytical methods to mitigate the potential bias risks. The eligibility criteria and utilization of the StArt software also played significant roles in minimizing biases.

## 3 Results and Discussion

### 3.1 Criteria Selection and Main Information

**Figure 2:**
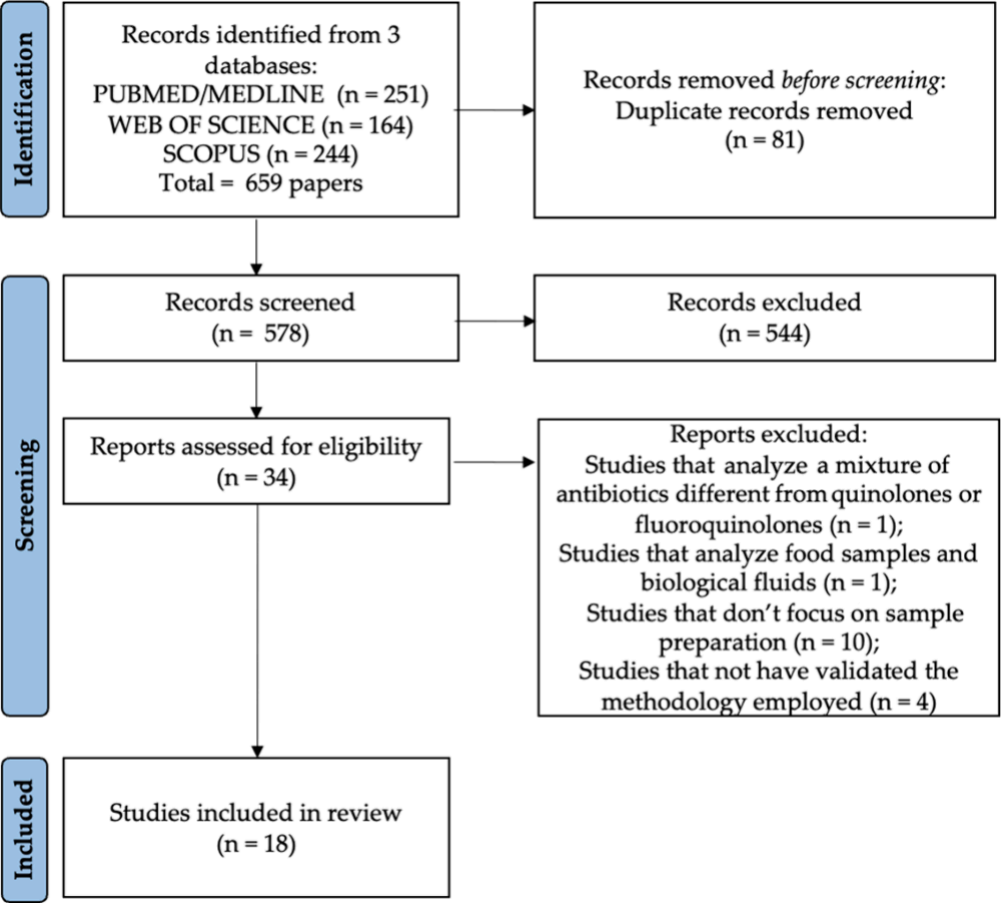
Flowchart of the systematic review employing Prisma diagram.

The search strategy retrieved a total of 659 publications from three databases: PubMed/Medline (*n* = 251), SCOPUS (*n* = 244), and Web of Science (*n* = 164). After 81 duplicates were eliminated, 578 articles remained. These selected articles then underwent a screening process involving the reading of titles, keywords, and abstracts. The screening was independently conducted. Subsequently, 34 relevant studies were identified. In the next phase, the full text of these selected articles underwent a thorough review, resulting in the exclusion of 16 studies that did not meet the eligibility criteria. The detailed selection process of the included articles in this review is visually presented in Figure 2.

Sixteen articles were excluded based on the following criteria: studies that analyze a mixture of antibiotics different from quinolones or fluoroquinolones (1); studies that analyze food samples and biological fluids (1); studies that do not focus on sample preparation (10); studies that do not have method validation for the methodology employed (4). Various analytical methods (totaling 18 studies) for the determination of quinolones and fluoroquinolones in aqueous matrixes were employed in the selected studies.

### 3.2 Occurrence Quinolones and Fluoroquinolones

**Figure 3:**
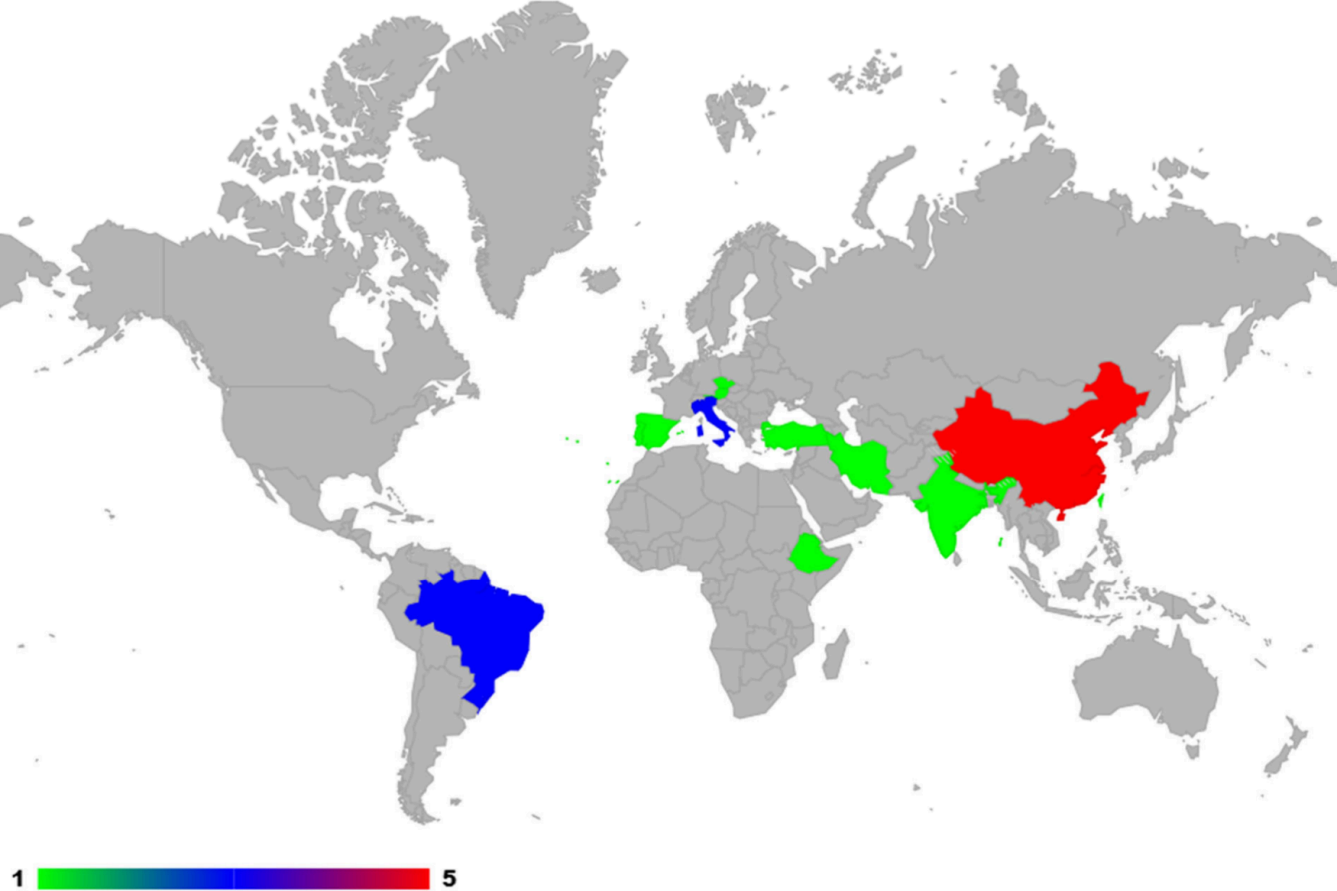
Countries where studies on the occurrence of quinolones and fluoroquinolones in aquatic environments were carried out.

Table 2 summarizes the main obtained data, providing information on the analytes, sample preparation technique, instrumental technique, and country where the study was developed the study. Among the included articles, it is noteworthy that investigations into the analysis of quinolones and fluoroquinolones in aqueous media using sample preparation have been conducted in various countries worldwide (as illustrated in Figure 3). However, this review emphasizes the significant contribution of China, with five publications. It is also relevant to highlight the collaboration among countries in conducting these studies, such as Turkey, Czech Republic, and Austria, as well as Italy and India, indicating scientific cooperation in research related to the topic.

**Table 2:** Summary of Determination of Quinolones and Fluoroquinolones in Aqueous Matrixes^a^

Analyte	Sample preparation	Instrumental technique	Country	Reference
**Methods Employing LLE and Its Miniaturized Variations**				
CINO, OXO, NALI, FLUME	HF-LPME with voltage (100 V)	HPLC–MS/MS	Taiwan	Wang & Wang, 2012 (^48^)
DIFL, FLU, NAL, PEF, LOM, MAR, CIP, ENR, FLE, ORB, MOX, ENO, LEV, DAN, PIP, CIN, NOR, SAR	SALLE	UHPLC–MS/MS	Spain	Lombardo-Agüí et al., 2014 (^49^)
LEV, NOR, DAN	LDS-UA-LLME	Electroanalysis	Brazil	De Oliveira & Trindade, 2016 (^50^)
CIP	LLME	Electroanalysis	Brazil	Gabbana et al., 2018 (^51^)
CIP	SALLE	HPLC–DAD	Ethiopia	Gezahegn et al., 2019 (^34^)
NOR	LLME	Electroanalysis	Brazil	Rosa et al., 2019 (^52^)
OFL, NOR, CIP, ENR	hDES-SA-LLME	HPLC–UV	Korea	Li et al., 2020 (^40^)
CIP, LEVO, LOM, ENR, MOX	LIS-automated SDME	HPLC–FD	Turkey, Czech Republic, and Austria	Yildirim et al., 2022 (^35^)
**Methods Employing SPE and Its Miniaturized Variations**				
ENR, MAR, FLE, LOM, SPA	MSPE	HPLC–DAD	China	Huang et al., 2013 (^41^)
CIP, ENR, LEVO, MAR, NOR	SPE	HPLC–FD	Italy	Speltini et al., 2015 (^44^)
OFL, CIP, ENO, PEF	PMME	HPLC–DAD	China	Liu et al., 2015 (^42^)
MAR e ENR	SPE	HPLC–FD	Italy and India	Speltini et al., 2016 (^45^)
MAR, NOR, CIP, LOM, ENR, SPA, SAR	MSPE	HPLC–DAD	China	Liu et al., 2016 (^43^)
CIP, DANO, ENR, LEVO, MAR	SPE	HPLC–FD	Italy	Speltini et al., 2017 (^46^)
NOR, CIP, ENO, LOM	MSPE	HPLC–UV	China	Tong et al., 2017 (^38^)
NOR, CIP, ENR	MSFIA-SPE	HPLC–FD	Portugal	Peixoto et al., 2018 (^47^)
FLE, ENO, NOR, CIP, ENR, LOM	MSPE-DLLME	HPLC–UV	China	Fan, Zheng, Ma, 2020 (^39^)
OFL, CIP, ENR, MOX	MSPE	HPLC–UV	Iran	Bayatloo et al., 2022 (^33^)

a CIP, ciprofloxacin; ENR, enrofloxacin; NOR, norfloxacin; MAR, marbofloxacin; LOM, lomefloxacin; LEV, levofloxacin; ENO, enoxacin; OFL, ofloxacin; MOX, moxifloxacin; FLE, fleroxacin; DAN, danofloxacin; SPA, sparfloxacin; FLU, flumequine; CIN, cinoxacin; PEF, pefloxacin; SAR, sarafloxacin; DIF, difloxacin; NAL, nalidixic acid; ORB, orbifloxacin; PIP pipemidic acid; OXO, oxolinic acid; LLME, liquid–liquid microextraction; SDME, single-drop microextraction; DLLME, dispersive liquid–liquid microextraction; MSFIA-SPE, multisyringe flow injection analysis coupled to solid-phase extraction; UA-LLME, ultrasound-assisted liquid–liquid microextraction; LPME, liquid-phase microextraction; PMME, polymer monolith microextraction; MSPE, magnetic solid-phase extraction.

Quinolone antibacterial agents exhibit a broad spectrum of action against various pathogens and can be employed for treating urinary tract infections, digestive tract and respiratory tract infections, sexually transmitted diseases, and skin and infections of bones and joints. They are also utilized in infections in animals.^27^ A total of 21 quinolone antibacterial agents were investigated in the selected studies, with these compounds being utilized in both clinical and veterinary practices. Table S1 displays the chemical structures, molecular mass, chemical formula, and log *P* and p*K*_a_ (predicted properties) of these substances.

**Figure 4:**
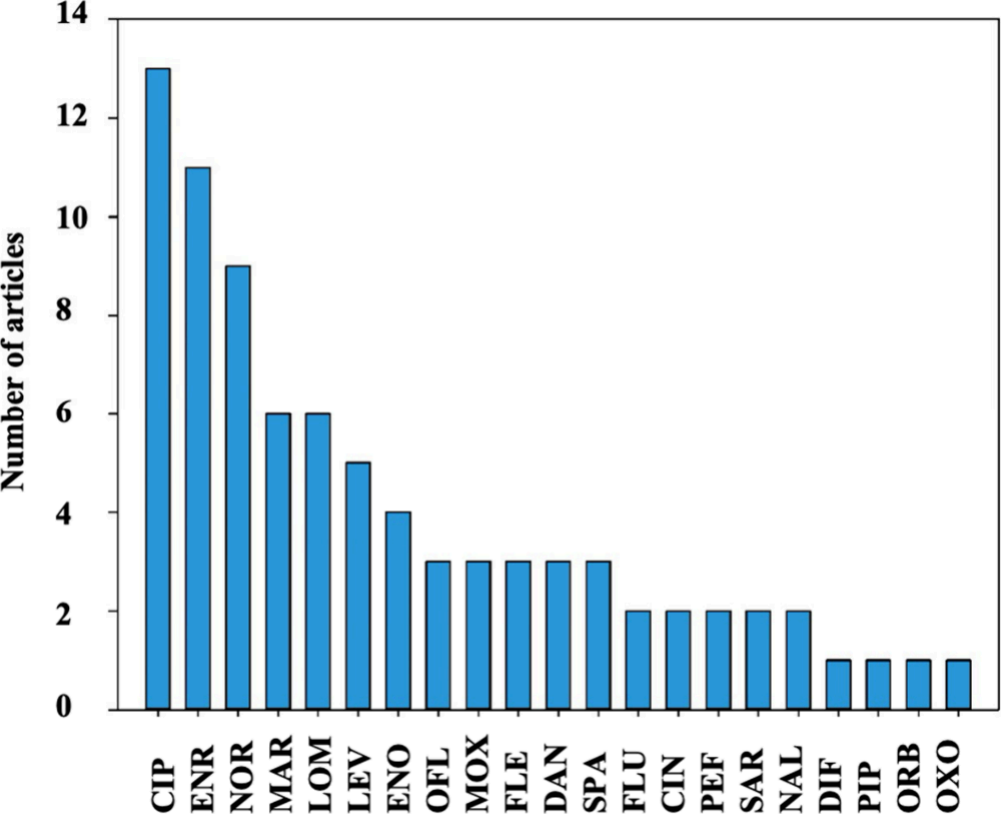
Quinolonic and fluoroquinolonic antibacterials evaluated in select studies. CIP, ciprofloxacin; ENR, enrofloxacin; NOR, norfloxacin; MAR, marbofloxacin; LOM, lomefloxacin; LEV, levofloxacin; ENO, enoxacin; OFL, ofloxacin; MOX, moxifloxacin; FLE, fleroxacin; DAN, danofloxacin; SPA, sparfloxacin; FLU, flumequine; CIN, cinoxacin; PEF, pefloxacin; SAR, sarafloxacin; NAL, nalidixic acid; DIF, difloxacin; PIP, pipemidic acid; ORB, orbifloxacin; OXO, oxolinic acid.

In the selected studies, the most frequently evaluated quinolones and fluoroquinolones were ciprofloxacin (CIP) in 72.2% of the studies, enrofloxacin (ENR) in 61.1%, norfloxacin (NOR) in 50%, marbofloxacin (MAR) and levofloxacin (LEV) in 33.3%, as illustrated in Figure 4. CIP and ofloxacin (OFL) that are second-generation quinolones have been widely used against the bacterium *Pseudomonas aeruginosa*, which is a microorganism causing a variety of infections, especially in individuals with compromised immune systems or in hospital environments.^28^

In China, CIP is the most widely used fluoroquinolone in humans and swine production, where ENR and NOR are also extensively employed.^27^ In contrast, in Ethiopia, CIP ranks among the top 10 antibacterial agents frequently used in the country.^29^ In Brazil, a study revealed that CIP and LEVO were the most consumed fluoroquinolones between 2013 and 2016.^30^ Similar findings have been observed in the literature,^31^ where these pharmacological agents were the most used among fluoroquinolones in a university hospital in Italy from 2008 to 2014. The report on antibiotic use in Italy also supports the idea that CIP and LEV are the most prevalent fluoroquinolones in the country. In this context, a connection is noted between the selected fluoroquinolones for water analysis and the conducted studies, as the evaluated fluoroquinolones are those that could indeed be contaminating aquatic environments.

Furthermore, it is worth noting that, of the 18 selected studies, 11 assessed the presence of enrofloxacin in water. This emphasis can be attributed to the imperative need to monitor the occurrence of this compound in water resources, as issues related to human safety arise due to the known toxic effects of enrofloxacin in humans, for which its use is prohibited. Additionally, attention directed toward the presence of this antimicrobial in water also extends to certain livestock, such as poultry, given its potential capacity to induce selective pressure on organisms, favoring the development of resistance to fluoroquinolones, which is a cause for great concern.^27^ Due to the widespread use of quinolones, they and their metabolites often enter the environment in their active form, contributing to bacterial resistance and causing toxic effects on fauna and flora with severe impacts on aquatic ecosystems. Therefore, reducing the release of these substances into the environment is of great strategic importance.^32^

**Figure 5:**
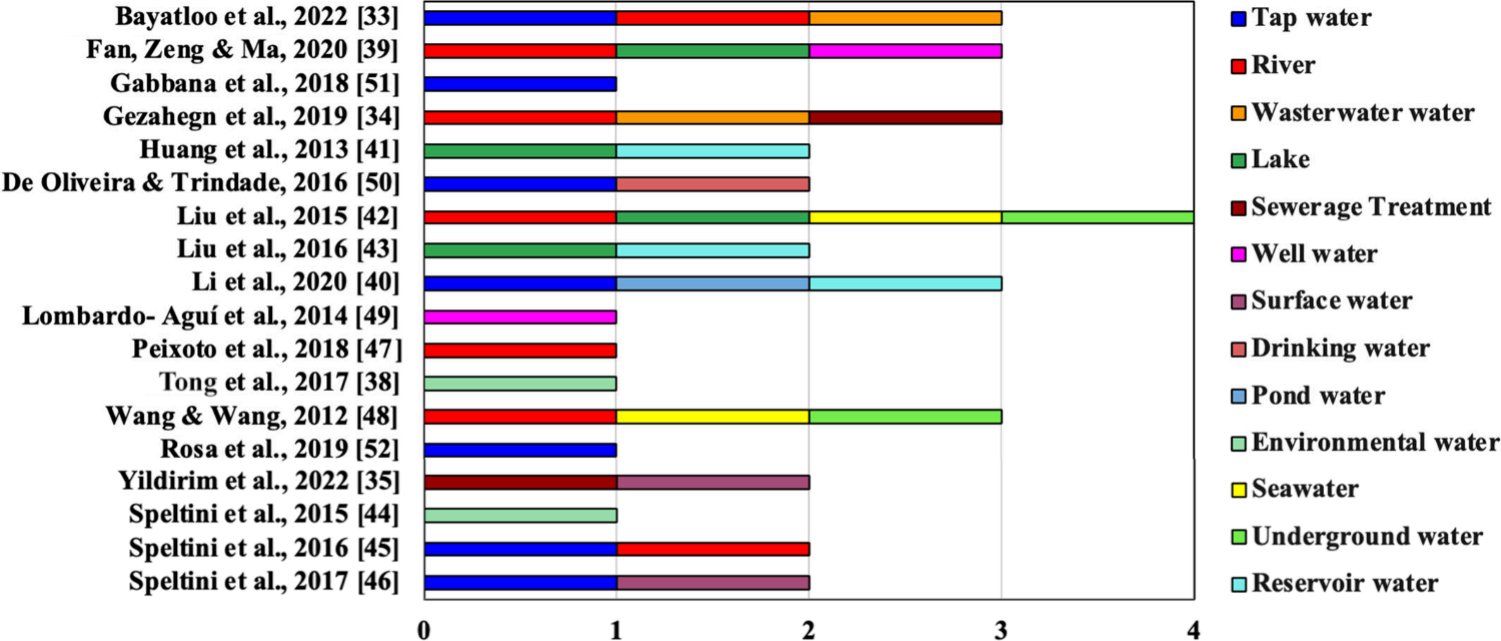
Sources of aqueous matrixes analyzed in the selected studies.

Another relevant aspect pertains to the diversity of aqueous matrixes investigated, encompassing samples derived from pharmaceutical industry wastewater,^33,34^ hospital wastewater,^34^ as well as residual wastewater.^35^ Additionally, potable and contaminated water from various locations were examined; Figure 5 summarizes the obtained data.

In this context, it is important to highlight the significance of sample preparation methods that are appropriate for the studied conditions and to take into account the physicochemical properties of fluoroquinolones. Aqueous matrixes can exhibit substantial variation in their chemical composition, as well as in pH, and may contain a wide range of interfering substances that can coelute, thereby affecting the selectivity and sensitivity of the analytical method. The matrix effect, which refers to the interference of other components present in the sample with the analytical signal of the target analytes, can influence the accuracy and precision of the results.^36^ Furthermore, fluoroquinolones have a high affinity for sludge, soil and sediments, which can directly influence the determination of these compounds in aqueous samples with a high load of these components.^37^

Another point worth highlighting is that studies show the concentration of fluoroquinolones varies depending on where the sample is collected, often being found in greater abundance in wastewaters.^37^ Consequently, tailoring the analytical procedures to account for these matrix variations is crucial to ensuring both the accuracy and the reliability of the results.

### 3.3 Instrumental Techniques

These pieces of information help identify the areas where these fluoroquinolones are most common, which makes it possible to analyze the compounds’ traces. Furthermore, because the study is based on real samples, they enable the evaluation of the applicability and accuracy of the employed technique.^33^

Regarding the analytical techniques employed in the selected studies, High-Performance Liquid Chromatography (HPLC) stands out as the predominant method for the separation and identification of these compounds, owing to the nonvolatility characteristic present in most pharmaceutical products.^34^ Additionally, it is a technique that boasts high resolution, sensitivity, and versatility, providing rapid and accurate analyses. Over the past 40 years, HPLC has emerged as the most advanced analytical technique, widely disseminated, and adopted in analysis laboratories, especially in the chemical and pharmaceutical industries, medical sectors, and various scientific fields, including governmental agencies.^33,34^ Based on this, 14 works utilized High-Performance Liquid Chromatography (HPLC) being four with ultraviolet detection (UV),^33,38−40^ four with diode array detection (DAD),^34,41−43^ four with fluorescence detection (FD),^35,44−47^ one with mass spectrometry detection (MS)^44,48^ and only one employed Ultra-High-Performance Liquid Chromatography coupled to mass spectrometry detection (UHPLC-MS/MS).^49^ Other three works employed electroanalysis.^50−52^

Another point of relevance is the influence of different types of coupled detectors, enabling the determination of antibacterials with variations in the sensitivity and specificity. HPLC-UV or HPLC-FD is the most employed analytical technique for quinolones/fluoroquinolones determination. However, mass spectrometry has emerged as the most effective in analyses, demonstrating analytical superiority compared to other available detectors, as it allows for the precise identification of components through the analysis of their molecular masses, enabling reliable analysis of complex mixtures; nevertheless, only two selected studies employed MS detection.^48,49^

### 3.4 Sample Preparation Procedures

#### 3.4.1 Overview

It is important to mention that the sample preparation under study is crucial for analyses, especially when dealing with complex matrixes and analytes that are highly diluted in samples to ensure higher sensitivity. Its purpose is to remove as many interferents from the matrix as possible, concentrate, and extract the analyte. Quinolones/fluoroquinolones are commonly detected in trace concentrations in aqueous matrixes, justifying the need for the preconcentration step.^33,40^

Among the selected studies in this review, 44% employed liquid–liquid extraction (LLE) techniques and its miniaturized versions, while 56% opted for solid-phase extraction (SPE) and its miniaturized variations. SPE is widely used in relation to LLE due to several advantages, such as greater selectivity in analyte extraction, allowing more effective interaction with the solid-phase material, a larger sample volume can be used, resulting in greater preconcentration of the analyte.^36^ Furthermore, SPE uses significantly lower volumes of organic solvents, making the process more sustainable and cost-effective.^53^

It is noteworthy that a significant portion of the literature used in this review utilized miniaturized variations of conventional techniques, SPE and LLE, such as liquid–liquid microextraction (LLME),^52^ hydrophobic deep eutectic solvent coupled with shaker-assisted liquid–liquid microextraction (hDES-SA-LLME),^40^ single-drop microextraction (SDME),^35^ liquid–liquid microextraction (LLME),^51,52^ ultrasound-assisted liquid–liquid microextraction (UA-LLME),^49^ liquid-phase microextraction (LMPE),^48^ polymer monolith microextraction (PMME),^42^ magnetic solid-phase extraction (MSPE),^33,41,43^ MSPE-LLME,^39^ and salting-out assisted liquid–liquid extraction (SALLE).^34,49^ Therefore, out of the 18 selected studies, seven employed miniaturized techniques, highlighting the growing concern within the scientific community to develop and enhance sample preparation methods, aiming for faster, economically efficient extractions. This scenario drives the simplification and miniaturization of procedures, seeking more sustainable and eco-friendly techniques characterized by reduced sample usage and minimized utilization of materials and organic solvents, contributing to the construction of a more sustainable future.^23^

Sample preparation also requires the optimization of various parameters, which vary according to the employed technique, whether LLE or SPE, aiming to ensure the efficiency of the extraction process to achieve trace levels of the analytes. Information from selected studies related to the optimization of parameters involving the LLE and its miniaturized variations and SPE and some other miniaturized techniques is summarized in Tables 3 and 4, respectively. This systematization aims to provide a consolidated and comparative view of the conducted experiments, contributing to a more comprehensive understanding of the methods adopted in the studies.

**Table 3:** Optimization Parameters of Studies Involving LLE and Its Miniaturized Variations^a^

Sample preparation	Sample volume/mL	Extractor solvent/amount	pH	Type and amount of salt	Stirring	Extraction time/min	Reference
HF-LPME with voltage (100 V)	4	Extractor: dipping a 7 cm fiber into 2-octanone by 10 s	2.0		750 rpm	20	Wang & Wang, 2012 (^48^)
		Acceptor: 40 mM borate buffer pH 10/25 μL					
SALLE	5	5% formic acid in acetonitrile/10 mL	7.0	1 g of NaCl	9000 rpm for 5 min		Lombardo-Agüí et al., 2014 (^49^)
		4 g of MgSO_4_					
LDS-UA-LLME	12	Acetone/25% (v/v)	10.0	NaCl 25% (m/v)	3000 rpm for 5 min	25	De Oliveira & Trindade, 2016 (^50^)
LLME	25	choline chloride: malonic acid (1:1, molar ratio)/120 mg	6.0		700 rpm for 5 min		Gabbana et al., 2018 (^51^)
SALLE	10	Acetonitrile/5 mL	3.0	MgSO_4_ (4 g)	4000 rpm for 5 min	6	Gezahegn et al., 2019 (^34^)
LLME	50	[HMIM][PF6]/25.54 mg	5.0		700 rpm for 3 min		Rosa et al., 2019 (^52^)
hDES-SA-LLME	10	*In situ* formed hDES composed of thymol: heptanoic acid (2:1, molar ratio)/100 μL	4.0–7.0		2 min	8	Li et al., 2020 (^40^)
LIS-automated SDME	3	NADE (thymol: hexanoic acid, 1:3, molar ratio)/60 μL	7.0		4000 rpm for 3 min	16	Yildirim et al., 2022 (^35^)

a LDS-UA-LLME, low-density solvent and ultrasound-assisted liquid–liquid microextraction; LLME, liquid–liquid microextraction; SALLE, salting-out assisted liquid–liquid extraction; hDES-SA-LLME, hydrophobic deep eutectic solvent shaker-assisted liquid–liquid microextraction; [HMIM][PF6], 1-hexyl-3-methylimidazolium hexafluorophosphate; LIS-automated SDME, lab-in-syringe automated direct immersion single drop microextraction; NADE, natural deep eutectic solvent.

**Table 4:** Optimization Parameters of Studies Involving SPE and Its Miniaturized Variations^a^

Sample technique	Adsorbent/amount	Eluent solvent	Eluent volume	pH	Extraction time/min	Desorption time/min	Reuse/times	Sample volume/mL	Reference
MSPE	Nanosized spherical magnetic poly(vinylimidazole-*co*-divinylbenzene) particles (Fe_3_O_4_@SiO_2_@P(VI-*co*-DB))/50 mg	Methanol: acetic acid (96:4, v/v)	500 μL	6.0	30	30		50	Huang et al., 2013 (^41^)
SPE	Reduced graphene oxide–silica (RGO-silica)/ 200 mg	Acetonitrile: 50 mM tetrabutyl ammonium hydroxide (30:70, v/v)	5 mL	7.0–7.7			10	500	Speltini et al., 2015 (^44^)
PMME	ZnO@poly(methacrylic acid-*co*-ethylene dimethacrylate)	0.1% trifluoracetic acid in acetonitrile	0.05 mL min^–1^	7.0				0.8	Liu et al., 2015 (^42^)
SPE	pyrolized lignin-silica 0.7% (LG-silica)/200 mg	Acetonitrile: 50 mM	4 mL	∼7.5			3	50	Speltini et al., 2016 (^45^)
		tetrabutylammonium hydroxide (30:70, v/v)							
MSPE	Boronic acid functionalized magnetic nanoparticles modified with poly(4-vinylphenylboronic acid-divinylbenzene)/30 mg	Methanol/0.5% formic acid aqueous (85:15, v/v)	500 μL	8.0	12	5	30	50	Liu et al., 2016 (^43^)
SPE	silica-supported graphitic carbon nitride (g-C_3_N_4_@silica)	25 mM H_3_PO_4_ aqueous solution-acetonitrile (80:20)	6 mL	7.5–8.0			4	50	Speltini et al., 2017 (^46^)
SPE	MMIP based on acrylamide as functional monomer and *N,N*′-methylenebis(acrylamide) as cross-linked and Fe_3_O_4_/20 mg	Methanol: water (60:40, v/v) + 0,1% formic acid		7.0	20	15	8	50	Tong et al., 2017 (^38^)
MSFIA-SPE	SupelMIP SPE Fluoroquinolones/25 mg	Methanol: ammonium hydroxide (98:2, v/v)	1500 μL	7.2				100	Peixoto et al., 2018 (^47^)
MSPE-DLLME	MIP based on magnetic graphene oxide embellished with mesoporous silica modified with vinyl groups (VTTS-MGO@ mSiO_2_)/20 mg	Tetrachloroethane	50 μL	6.0	12	6	5	50	Fan, Zheng, Ma, 2020 (^39^)
MSPE	Maltodextrins nanosponges and Fe_3_O_4_/5 mg	Methanol: water (1:1, v/v) containing 1% triethylamine (v/v)	300 μL	6.0	25	10	7	35	Bayatloo et al., 2022 (^33^)

a MSPE, magnetic solid-phase extraction; SPE, solid-phase extraction; PMME, polymer monolith microextraction; MSFIA-SPE, multisyringe flow injection analysis coupled to solid-phase extraction; DLLME, dispersive liquid–liquid microextraction.

Overall, it is interesting to note the variety of methods, materials, and solvents employed, most of which are accessible, that can be applied to the analysis of various fluoroquinolones/quinolones in water across different aquatic environments. Additionally, there is a growing interest in developing effective analytical processes capable of quantifying trace amounts of fluoroquinolone and quinolone compounds through simpler, cheaper, and more efficient methods, thereby enabling a broader assessment of pollution in aquatic environments.^54^

In addition, the validation parameters were compiled, showing data within the limits established for the most diverse validation guides followed (Table S2). As expected, methods employing fluorescence detection and mass spectrometry achieved the best limits of quantification. Values ranged between 0.01 and 0.051 ng mL^–1^ for fluorescence detection and between 0.02 and 2.0 ng mL^–1^ for the different analytes. For example, a method for the determination of 19 fluoroquinolones in water by UHPLC–MS/MS, reaching detection limits between 0.010 and 0.090 ng mL^–1^, among them NOR, CIP and ENR with limits of detection of 0.04, 0.04, and 0.02 ng mL^–1^, respectively, has been developed.^49^ Other work reached the limit of detection of 3 ng mL^–1^ for the same analytes using HPLC-UV.^40^ Lower detection limits at trace levels can enhance the early identification of contaminants, enabling environmental monitoring and mitigating risks to public health and the environment. This improved sensitivity allows for the implementation of corrective measures before contamination reaches critical levels.^54^

In its traditional form, SPE uses cartridges loaded with an adsorbent material, usually C18;^45^ however, various other materials have been explored,^38,39,47^ such as selective adsorbent materials, namely, molecularly imprinted polymers (MIPs), using different sample preparation techniques as MSPE followed to DLLME,^39^ MSPE,^39^ multisyringe flow injection analysis coupled to solid-phase extraction (MSFIA-SPE).^47^ In addition, magnetic adsorbent based on poly(vinylimidazole-*co*-divinylbenzene)^41^ and monolith based on poly(methacrylic acid-*co*-ethylene dimethacrylate) functionalized with zinc oxide nanoparticles^42^ have been synthesized.

The literature highlights a broad diversity of reported adsorbents, underscoring the significance of selecting the most appropriate adsorbent for a specific analytical objective. In this context, considerations such as cost, solvent choice, and overall methodology must be carefully weighed. In addition, sustainability must be considered, especially in large-scale applications.

#### 3.4.2 LLE and Its Miniaturized Variations

In LLE, which is a method that entails the partitioning of a compound between two immiscible liquids or phases, each possessing distinct solubilities for the compound, some characteristic parameters include the appropriate selection of the nature and volume of extracting (and dispersing solvents), pH, agitation, and many times centrifugation time to promote the transfer of analytes and clear phase separation at the end of the process.^21,22^

From the analyzed studies, it can be observed that various extracting solvents were employed in addition to a significant variation in the pH of samples. This possibility is because quinolones and fluoroquinolones exhibit amphoteric acid–base properties (see Table S1). In aqueous matrixes, quinolones and fluoroquinolones can exist in various forms including cationic, anionic, or intermediate states. This diversity is attributed to the presence of a carboxylic group and the charged amino group associated with the piperazine moiety, which influence the compounds’ chemical behavior and interactions.^55^

Thus, pH control is essential to improve separation and consequently achieve a higher detection rate. Three works described more acid conditions (pH 2–3) to extract the analytes,^34,48,51^ while the other three used pH = 7 (or neutral)^35,40,49^ and only one work extracted the analytes in basic conditions (pH = 10).^50^ The addition of organic acids to the extraction solution promotes the presence of quinolones in a molecular state and may significantly increasing their partition coefficients in the organic phase, and then improving the recovery of analytes.^56^ These choices are related to the entire method employed, considering the influence of each parameter on the final recovery.

It is worth noting that studies conducted under more acidic conditions require the water sample pH to be lower than the p*K*_a_ of the analytes.^34,40,48,52^ This ensures that the carboxylic group of the analytes is in its nonionized form and that the piperazine nitrogen is protonated. As a result, there is an increased affinity for the organic phase, leading to a higher partition coefficient and greater extraction efficiency.^34,47^ Furthermore, the pH variations observed in these studies are directly related to the p*K*_a_ values of each fluoroquinolone. Also, pH variations influence the possible electrostatic interactions that may occur between the selected solvent and the analyte in question.^51^

Another important factor to consider in LLE and its miniaturized variations is the addition of salt to samples. The salting-out effect plays a significant role in modifying the physicochemical properties of the system. Introducing saline ions, influences the partitioning of analytes between the aqueous and organic phases, thereby optimizing the extraction efficiency.^34^ However, it is important to emphasize that different salts and concentrations will cause different degrees of phase separation, requiring optimization to obtain separation efficiency.^34^ The presence of salt, based on its ionic effect, can enhance the selectivity of the process, leading to a higher recovery of target compounds. From the studies included in this review, it was observed that the ions addition, such as sodium chloride,^49,50^ and magnesium sulfate,^36,49^ provided a high extraction efficiency of fluoroquinolones compared to other ions evaluated. Thus, understanding the impact of saline concentration in LLE is crucial for the controlled manipulation of intermolecular interactions, providing an effective means to adjust extraction efficiency.^57^

The LLE exhibits advantageous characteristics, notably simplicity; in its standard configuration, a separation funnel is employed, utilizing a broad spectrum of commercially available solvents, thereby offering an extensive range of solubilities and selectivity. Nevertheless, this technique is not devoid of drawbacks. Samples with a high affinity for water (polar compounds) may undergo partial extraction by the organic solvent, resulting in analyte loss. The propensity for emulsion formation entails a significant time consumption, as relatively large volumes of both samples and solvents are required, posing disposal challenges.^58,59^ Additionally, the process proves relatively challenging to automate, but alternatives are already presented, such as for LLE classical^60^ and DLLME.^61^

Despite the mentioned drawbacks, LLE continues to be regarded as a classical sample preparation technique. Since the advent of “green analytical chemistry”, the pursuit of reducing organic solvent consumption has intensified. Miniaturization emerges as an effective strategy to meet this requirement. In this review, it is noted that studies involving LLE employed some miniaturization techniques, indicating that the scientific community has sought new alternatives to conventional LLE. In comparison to conventional LLE, microextraction techniques demonstrate a significant reduction in the ratio between the volume of organic solvent and the sample solution, ensuring high enrichment factors for extraction and, consequently, standing out as attractive approaches in trace analysis.^21^

#### 3.4.3 SPE and Its Miniaturized Variations

The SPE is a versatile technique applicable for various purposes, such as analyte extraction and/or concentration, analyte isolation, sample cleanup, and even storage. It presents several advantages, such as high recovery efficiency, reduced consumption of organic solvents compared to the solvent extraction technique, shorter preparation time, ease of operation, and improved automation.^20^ The efficiency of the extraction process can be influenced by various parameters employing SPE, including the appropriate selection of the sorbent and its quantity, choice of the elution solvent and its volume, washing conditions, sample pH value, and ionic strength. Additionally, sample volumes, the total analysis time, and the reusability of the materials employed play significant roles in the optimization and enhancement of the extraction technique.^62^

Because sample pH affects both the material’s and the analyte’s charge, it plays a crucial role in adsorption and, by extension, analyte recovery. It has been noted that, in studies that used SPE, the pH range that was used to prepare samples was between 6 and 8. This is due to the necessity of efficient electrostatic interactions between the materials used in SPE and the quinolones and fluoroquinolones, which are influenced by pH control, as it affects the degree of ionization, polarity, water solubility, and extractability. In general, fluoroquinolones exhibit the lowest solubility in water around pH 7–8 since they are at a state of equilibrium between their zwitterionic and neutral forms.^27^

Another crucial point is the reutilization of adsorbent, such as an adsorbent based on boronic acid functionalized magnetic nanoparticles modified with poly(4-vinylphenylboronic acid-*co*-divinylbenzene), which stands out due to its remarkable reusability, allowing its application for up to 30 cycles (Table 4).^43^ This emphasis underscores the significance of this study, highlighting the economic efficiency concerning synthesis reagents and the substantial reduction in the generated waste volume. This illustrates a scientific approach to sustainable environmental practices.

SPE involves several steps, including sorbent conditioning, where the sorbent is moistened to activate functional groups, followed by sample percolation through the sorbent, washing with a low elution strength solvent to remove potential interferents, and finally, elution of the analyte with an appropriate solvent. However, it has the disadvantage of memory effects when the SPE column is reused, leading to progressive sorbent deterioration. The traditional *offline* mode involves sample preparation outside the instrumental system, with the prepared sample subsequently being introduced into the system for detection. This procedure involves multisteps and high cost and is relatively time-consuming, which may result in a high error rate, unstable recovery rate, and require bulky solvents and loading samples. An advantage of SPE procedures is the possibility of using the *online* mode, in which the extraction occurs within the instrumental system, offering advantages such as high sample throughput, greater precision, and reduced reagent consumption.^63^

## 4 Conclusions

This review has several strengths, with a comprehensive approach in analyzing studies that employed various analytical methods involving SPE/LLE and its miniaturized variations in sample preparation. It is important to note that the final extraction efficiency relies on the entire process, from selecting the instrumental technique with appropriate detection limits to going through the entire sample preparation to achieve a good recovery through adjustments in the extraction parameters. Additionally, the selected works investigated quinolones and fluoroquinolones of global interest, which are correlated with the presence of certain bacterial resistance genes, primarily in aquatic environments. The methodology adopted for selecting the studies was robust, incorporating multiple eligibility criteria and utilizing a computational tool, StART, contributing to increased reliability and reducing the risk of bias in the article selection process. However, this review also has some limitations. The research was confined to only three databases and the inclusion of articles written in English may have resulted in the exclusion of potentially relevant studies. Additionally, some studies did not provide optimization-related data, making it challenging to draw certain comparisons between the methods used. The absence of this information can impact the comprehensive understanding and comparative assessment of the effectiveness of sample preparation techniques employed in the analyzed studies.

## Supporting Information Available

Tables containing chemical structures, molecular mass, chemical formula, log *P* and p*K*_a_ (predicted properties) and data of validation parameters of selected studies in this review (PDF)
