# Supplementary material for: Sample Preparation Approaches for Determination of Quinolones in Aqueous Matrixes: Systematic Review
Source: ACS Meas Sci Au. 2024 Nov 14;5(1):19–30. doi: 10.1021/acsmeasuresciau.4c00056 (PMC11843513; doi:10.1021/acsmeasuresciau.4c00056)
Supplement: Supplementary file 1 — tg4c00056_si_001.pdf [file tg4c00056_si_001.pdf]

Supporting Information

Sample preparation approaches for determination of quinolones in aqueous matrices: systematic review

Tainara Aparecida Nunes Ribeiro[1], Daiane Dulcileia Moraes de Paula[2], Marcella Matos Cordeiro Borges[2], Leandro Augusto Calixto[3], and Keyller Bastos Borges[2,*]

[1] Instituto de Física e Química, Universidade Federal de Itajubá (UNIFEI), Av. BPS, 1303, Pinheirinho, 37500-903, Itajubá, Minas Gerais, Brazil

[2] Departamento de Ciências Naturais, Universidade Federal de São João del-Rei (UFSJ), Campus Dom Bosco, Praça Dom Helvécio 74, Fábricas, 36301-160, São João del-Rei, Minas Gerais, Brazil.

[3] Departamento de Ciências Farmacêuticas, Instituto de Ciências Ambientais, Químicas e Farmacêuticas, Universidade Federal de São Paulo (UNIFESP), Campus Diadema, Prof. Artur Riedel, 275, Eldorado, 09972-270, Diadema, São Paulo, Brazil

Correspondence:

Prof. Keyller Bastos Borges, Ph.D., Departamento de Ciências Naturais, Universidade Federal de São João del-Rei, Campus Dom Bosco, Praça Dom Helvécio 74, Fábricas, 36301-160, São João del-Rei, Minas Gerais, Brazil, e-mail: keyller@ufsj.edu.br, Phone number: +55 32 3379 – 5163

Tables

Table S1. Chemical structures, molecular mass, chemical formula, logP and pKa (predicted properties).

Analytes	Chemical formula	Molecular mass /g	Chemical Structure	logP*	pK$_a$*
Ciprofloxacin (CIP)	$C_{17}H_{18}FN_3O_3$	331.3415	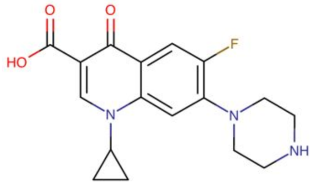	–0.86	pK$_{a1}$= 5.56<br>pK$_{a2}$= 8.77
Enrofloxacin (ENR)	$C_{19}H_{22}FN_3O_3$	359.3947	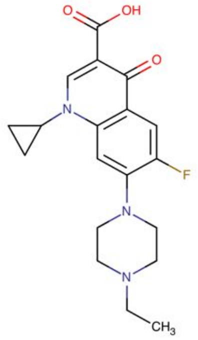	0.51	pK$_{a1}$= 5.55<br>pK$_{a2}$= 7.24
Norfloxacin (NOR)	$C_{16}H_{18}FN_3O_3$	319.3308	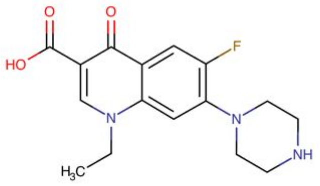	–0.97	pK$_{a1}$= 5.58<br>pK$_{a2}$= 8.77
Marbofloxacin (MAR)	$C_{17}H_{19}FN_4O_4$	362.3610	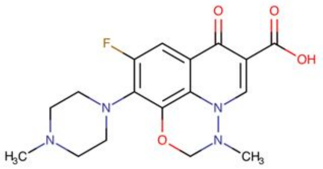	–0.53	pK$_{a1}$= 5.38<br>pK$_{a2}$= 6.16
Lomefloxacin (LOM)	$C_{17}H_{19}F_2N_3O_3$	351.3479	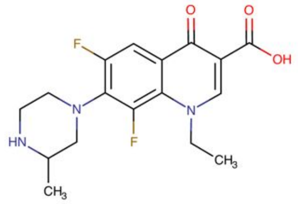	–0.43	pK$_{a1}$= 5.45<br>pK$_{a2}$= 8.78
Levofloxacin (LEV)	$C_{18}H_{20}FN_3O_4$	361.3675	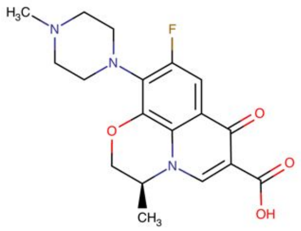	0.09	pK$_{a1}$= 5.35<br>pK$_{a2}$= 6.72
Enoxacin (ENO)	$C_{15}H_{17}FN_4O_3$	320.3189	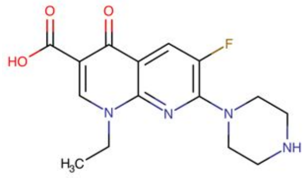	–1.00	pK$_{a1}$= 5.31<br>pK$_{a2}$= 8.68

Ofloxacin** (OFL)	$C_{18}H_{20}FN_3O_4$	361.3675	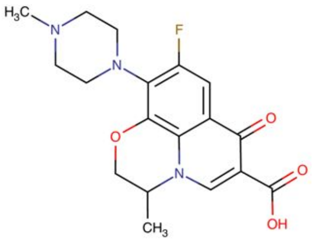	0.09	pK_{a1}= 5.35 pK_{a2}= 6.72
Moxifloxacin (MOX)	$C_{21}H_{24}FN_3O_4$	401.4314	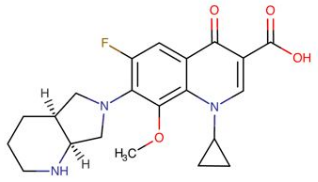	–0.51	pK_{a1}= 5.49 pK_{a2}= 9.51
Fleroxacin (FLE)	$C_{17}H_{18}F_3N_3O_3$	369.3440	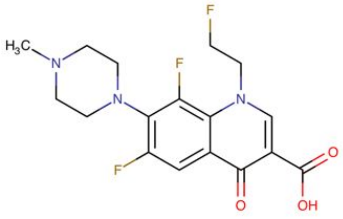	1.12	pK_{a1}= 5.44 pK_{a2}= 6.06
Danofloxacin (DAN)	$C_{19}H_{20}FN_3O_3$	357.385	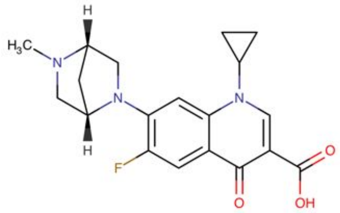	0.71	pK_{a1}= 5.65 pK_{a2}= 6.73
Sparfloxacin (SPA)	$C_{19}H_{22}F_2N_4O_3$	392.3998	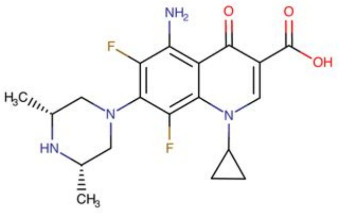	–0.081	pK_{a1}= 5.56 pK_{a2}= 8.88
Flumequine (FLU)	$C_{14}H_{12}FNO_3$	261.2484	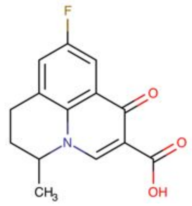	2.42	pK_{a1}= 5.81 pK_{a2}= –4.3
Cinoxacin (CIN)	$C_{12}H_{10}N_2O_5$	262.2182	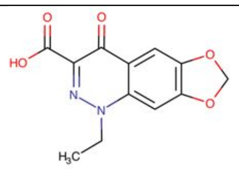	1.72	pK_{a1}= 4.93 pK_{a2}= –4.7
Pefloxacin (PEF)	$C_{17}H_{20}FN_3O_3$	333.3574	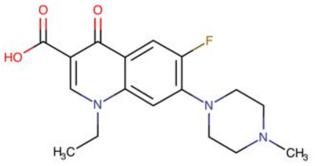	0.26	pK_{a1}= 5.55 pK_{a2}= 7.01

Sarafloxacin (SAR)	$C_{20}H_{17}F_2N_3O_3$	385.3641	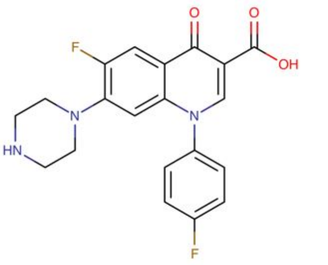	0.52	pK_{a1}= 5.55<br>pK_{a2}= 8.76
Difloxacin (DIF)	$C_{21}H_{19}F_2N_3O_3$	399.398	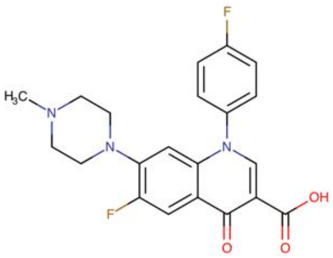	2.35	pK_{a1}= 5.64<br>pK_{a2}= 6.45
Nalidixic acid (NAL)	$C_{12}H_{12}N_2O_3$	232.2353	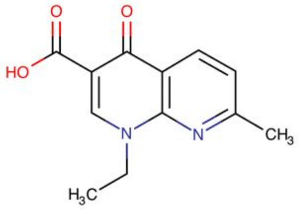	0.79	pK_{a1}= 4.37<br>pK_{a2}= 6.06
Orbifloxacin (ORB)	$C_{19}H_{20}F_3N_3O_3$	395.3820	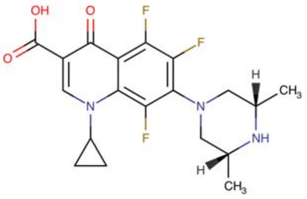	0.25	pK_{a1}= 5.49<br>pK_{a2}= 8.77
Pipemidic acid (PIP)	$C_{14}H_{17}N_5O_3$	303.3165	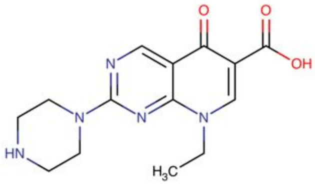	–1.8	pK_{a1}= 5.11<br>pK_{a2}= 8.66
Oxolinic acid (OXO)	$C_{13}H_{11}NO_5$	261.2301	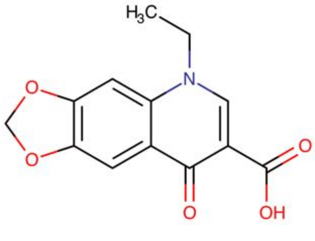	1.35	pK_{a1}= 5.39<br>pK_{a2}= –4.3

* All data were obtained from Chemaxon: pK_{a1} (Strongest Acidic) and pK_{a1} (Strongest Basic) are predicted properties;
** Ofloxacin is a racemic mixture, which consists of 50% levofloxacin (the biologically active component) and 50% enantiomer dextrofloxacin.

Table S2. Data of validation parameters of selected studies in this review.

Analytes	Instrumental technique	Linearity range / ng mL^{-1}	R^2	LOD / ng mL^{-1}	LOQ / ng mL^{-1}	Recovery / %	Reference
**Methods employing LLE and its miniaturized variations**							
CIN	HPLC–MS/MS	1–25	0.9993	0.45	1.51	90.8–109.6	Wang, Wang. 2011 (48)
OXO			0.9968	0.48	1.61		
NAL			0.9986	0.54	1.81		
FLU			0.9965	0.60	2.0		
SAR	UHPLC–MS/MS	0.07–20	0.999	0.02	0.07	73.6–107.8	Lombardo-Agüí, *et al.*, 2014 (49)
DAN		0.06–20	0.994	0.02	0.06		
CIP		0.15–20	0.996	0.04	0.15		
ENR		0.08–20	0.999	0.02	0.08		
DIFL		0.09–20	0.998	0.03	0.09		
FLU		0.02–20	0.998	0.01	0.02		
MAR		0.09–20	0.996	0.03	0.09		
OXO		0.03–20	0.994	0.01	0.03		
CIN		0.20–20	0.996	0.06	0.20		
ENO		0.19–20	0.998	0.06	0.19		
LOM		0.16–20	0.993	0.05	0.16		
NOR		0.12–20	0.994	0.04	0.12		
PIP		0.05–20	0.998	0.02	0.05		
ORB		0.05–20	0.998	0.02	0.05		
FLE		0.06–20	0.996	0.02	0.06		
PEF		0.31–20	0.995	0.09	0.31		
MOX		0.20–20	0.996	0.06	0.20		
LEV		0.12–20	0.997	0.04	0.12		
NAL		0.10–20	0.997	0.03	0.10		
LEV	Electroanalysis	4.8 – 31 (µmol L^{-1})	0.991	4.83 (µmol L^{-1})	-	-	De Oliveira & Trindade, 2016 (50)
NOR			0.996	2.64 (µmol L^{-1})			
DAN			0.996	2.92 (µmol L^{-1})			
CIP	Electroanalysis	1.66–9.94	> 0.97	0.83	-	71 – 93	Gabbana *et al.*, 2018 (51)
CIP	HPLC-DAD	0.1–100	0.9976	0.075	0.25	86–120	Gezahegn *et al.*, 2019 (34)
NOR	Electroanalysis	1.50–5.50 (µmol L^{-1})	0.99	0.52 (µmol L^{-1})	-	88–115	Rosa et al 2019 (52)
OFL	HPLC–UV	15-3000	1.000	3.0	9.0	84.9–103.6	Li *et al.*, 2020 (40)
NOR		15-1200	0.9998			93.7–115.3	
CIP		15-1200	0.9999			92.6–108.4	
ENR		30-1200	0.9997			92.6–1182	

LEV	HPLC–FD	0.1–5	0.9995	0.006	0.020	66.3	Yildirim *et al.*, 2022 (35)
CIP			0.9993	0.009	0.030	62.6	
LOM			0.9995	0.009	0.030	64.3	
ENRO			0.9991	0.006	0.020	52.7	
MOXI			0.9977	0.009	0.030	52.6	
**Methods employing SPE and its miniaturized variations**							
ENR	HPLC-DAD	2.0–200	0.9855	0.49	1.60	73.7–104.5	Huang *et al.*, 2013 (41)
MAR		2.0–200	0.9874	0.50	1.64	62.8–80.8	
FLE		5.0–200	0.9901	1.46	4.84	57.0–81.9	
LOM		1.0–200	0.9914	0.27	0.91	52.1–58.8	
SPA		1.0–200	0.9915	0.20	0.68	53.5–91.5	
CIP	HPLC–FD	1–250	> 0.9987	0.002	0.005	72–118	Speltini et al, 2015 (44)
ENR							
LEV							
MAR							
NEM							
OFL	HPLC-DAD	0.05−5	0.9949 –0.9982	0.007–0.034	0.026–0.115	70.7–108.3	Liu *et al.*, 2015 (42)
CIP							
ENO							
PEF							
MAR	HPLC–FD	0.020-1.0	0.9975	0.008	0.020	71–105	Speltini et al, 2016 (45)
ENR			0.9982				
NOR	HPLC-DAD	0.25–200	0.9933	0.051	0.17	72.0–118%	Liu *et al.*, 2016 (43)
CIP		0.50–200	0.9924	0.089	0.29		
MAR		0.25–200	0.9910	0.075	0.24		
LOM		0.50–200	0.9906	0.120	0.41		
ENR		0.25–200	0.9979	0.050	0.16		
SPA		0.25–200	0.9972	0.059	0.19		
SAR		0.25–200	0.9943	0.058	0.19		
CIP	HPLC–FD	1–50	> 0.9991	0.006	0.020	70–101	Speltini et al, 2017 (46)
DAN				0.006	0.020		
ENR				0.003	0.010		
LEV				0.003	0.010		
MAR				0.003	0.010		
CIP	HPLC–UV	10–5000	0.9914	7.2	24.0	70.6–103.6	Yukui *et al.*, 2017 (38)
LOM		10–5000	0.9926	5.8	19.3		

ENO		50–5000	0.9908	21.3	71.0		
NOR		10–5000	0.9904	4.1	13.7		
NOR	HPLC–FD	10–100 (pg)	*r* > 0.9993	0.013	0.035	74.6–95.6	Peixoto *et al.*, 2018 (47)
CIP				0.019	0.051		
ENR				0.006	0.017		
FLE	HPLC-UV	0.05–10	-	0.013	-	97.34–100.5	Fan; Zheng; Ma, 2020 (39)
ENO				0.012		92.18–99.53	
NOR				0.011		92.37–99.53	
CIP				0.012		95.18–99.74	
ENR				0.015		89.67–99.83	
LOM				0.015		94.26–99.65	
CIP	HPLC-UV	0.25–500	0.997–0.999	0.03	0.10	94.5–97.2	Bayatloo *et al.*, 2022 (33)
ENR							
OFL		0.50–500		0.06	0.20		
MOX							

CIP, ciprofloxacin; **ENR**, enrofloxacin; **NOR**, norfloxacin; **MAR**, marbofloxacin; **LOM**, lomefloxacin; **LEV**, levofloxacin; **ENO,** enoxacin; **OFL**, ofloxacin; **MOX**, moxifloxacin; **FLE**, fleroxacin; **DAN**, danofloxacin; **SPA**, sparfloxacin; **FLU**, flumequine; **CIN**, cinoxacin; **PEF**, pefloxacin; **SAR**, sarafloxacin; **DIF**, difloxacin; **NAL**, nalidixic acid; **ORB,** orbifloxacin; **PIP**, pipemidic acid; **OXO**, oxolinic acid.
